# Effects of interventional vascular embolization at different timing on prognosis and serum S100 calcium-binding protein B level of patients with aneurysmal subarachnoid hemorrhage

**DOI:** 10.4314/ahs.v23i3.65

**Published:** 2023-09

**Authors:** Peidong Yue, Lei Zhang, Bin Wang

**Affiliations:** 1 Department of Neurosurgery, Suzhou Hospital of Anhui Medical University (Suzhou Municipal Hospital of Anhui Province), Suzhou 234000, Jiangsu Province, China; 2 Department of Neurosurgery, The First Affiliated Hospital of Anhui Medical University, Hefei 230022, Anhui Province, China

**Keywords:** Aneurysmal subarachnoid hemorrhage, interventional vascular embolization, prognosis, S100 calcium-binding protein B, timing

## Abstract

**Objective:**

To evaluate the effects of interventional vascular embolization at different timing on the prognosis and serum S100 calcium-binding protein B (S100B) level of patients with aneurysmal subarachnoid hemorrhage (aSAH).

**Methods:**

A total of 229 aSAH patients enrolled from January 2016 to January 2020 were divided into an early-stage group (n=66), a middle-stage group (n=95) and a late-stage group (n=68. Their baseline data, serum indices and clinical outcomes were compared. The factors affecting their prognosis were analysed. The value of serum S100B level for predicting the prognosis was evaluated.

**Results:**

The early-stage group had the highest GOS score, and the late-stage group had the lowest score (P<0.05). Older age, large diameter of aneurysm, high Hunt-Hess grade upon admission, late surgical treatment and high S100B level were risk factors for the poor prognosis of aSAH patients. The optimal cut-off value of S100B for predicting the prognosis was 2.785 [µg/L. The area under the receiver operator characteristic curve, sensitivity, specificity, Youden index and 95% confidence interval were 0.892, 84.3%, 86.3%, 0.706 and 0.844-0.940, respectively.

**Conclusion:**

Early vascular interventional embolization is beneficial to the alleviation of brain injury and the reduction of serum S100B level.

## Introduction

As a common disease, cerebral aneurysm refers to aneurysm protrusion formed by the dilatation of weak cerebral artery wall due to increased vascular pressure. However, most patients have no obvious clinical manifestations or may not show symptoms all their lives. Only some patients with ruptured aneurysms suffer from subarachnoid hemorrhage (SAH) which has high morbidity and mortality rates. With the rapid development of minimally invasive surgery, vascular interventional surgery has become the first choice for treating SAH. Meanwhile, the timing of treatment is closely associated with the prognosis of patients^1^.

The level of serum S100 calcium-binding protein B (S100B) rises in the patients with brain injury induced by trauma, hemorrhage and ischemia, so S100B is often used as a biomarker for evaluating the severity of brain injury and prognosis^2^. However, the effects of different treatment timing of vascular intervention on the serum S100B level of SAH patients have seldom been referred. Therefore, we herein assessed the effects of early-, middle- and late-stage vascular interventional embolization on the prognosis and S100B level of patients with aneurysmal SAH (aSAH), aiming to provide references for clinical treatment.

## Materials and methods

### Subjects

A total of 229 aSAH patients enrolled from January 2016 to January 2020 were divided into three groups according to the timing of treatment, namely, an early-stage group (treated with interventional embolization within 3 d after admission, n=66), a middle-stage group (receiving interventionaembolization 3-10 d after admission, n=95) and a late-stage group (undergoing interventional embolization >10 d after admission, n=68).

The inclusion criteria involved: 1) Patients diagnosed as aSAH by computed tomography (CT), lumbar puncture, CT angiography and digital subtraction angiography, according to the diagnostic procedures and criteria in the *Chinese Guidelines for Diagnosis and Treatment of Subarachnoid Hemorrhage 2019^3^*, 2) those with single cerebral aneurysm and suffering from rupture and hemorrhage for the first time, 3) those having Hunt-Hess grading upon admission, 4) those undergoing vascular interventional embolization, and 5) those who were able to provide detailed general data and to finish follow-up. The exclusion criteria were as follows: 1) Patients with secondary SAH caused by other reasons, 2) those with dysfunction of vital organs or systems, autoimmune diseases or malignant tumors, 3) those with the history of trauma, other intracranial diseases, intracranial infection or other serious infectious diseases before onset, or 4) those who had rather severe&xa0;condition, died before operation or were unwilling to receive operation and left the hospital on their own. This study was reviewed and approved by the medical ethics committee of our hospital, and the patients or their families signed the informed consent.

### Treatment methods

After hospitalization, the patients should rest in bed, and their vital and neurological signs were monitored to ensure smooth breathing. Besides, they were given routine treatment such as dehydration, hemostasis, fluid infusion and nutritional support of cerebral nerves. Before operation, an intravenous micro-pump was established to infuse an appropriate amount of nimodipine, and femoral artery puncture was performed under tracheal intubation and general anesthesia, followed by the embolization of aneurysms with micro-coils and stents of appropriate specifications. Proper embolization and intact packing were ensured, and the parent arteries were kept unobstructed. After operation, the patients were given antiplatelet and anticoagulation therapy, and their lower limbs were strictly immobilized within 24 h.

### Baseline data

Age, gender ratio, smoking history, drinking history, hypertension history, location of ruptured aneurysm, diameter of aneurysm and Hunt-Hess grade were compared among the three groups of patients. According to the clinical manifestations, the patients were categorized into 5 grades using the Hunt-Hess scale, and a higher grade meant more serious disease. If severe vasospasm or systemic disease was disclosed by angiography, one additional point was added to the score.

### Detection of serum indices

Before operation and 1 week after operation, 5 mL of fasting peripheral venous blood was collected from all aSAH patients, and centrifuged at 3,000 rpm for 10 min. Then the supernatant was collected, and S100B, monocyte chemotactic protein-1 (MCP-1) and C-reactive protein (CRP) were detected using Human S100B Duo Set ELISA (DY1820-05, R&D Systems, USA), Human CCL2/ MCP-1 Quantikine ELISA Kit (DCP00, R&D Systems, USA) and Human C-Reactive Protein/CRP Quantikine ELISA Kit (DCRP00, R&D Systems, USA) strictly according to the instructions.

### Treatment outcomes and complications

Postoperative cerebral angiography was performed to observe the effect of embolization, and 100% embolization was defined as complete embolization, ≥90% embolization as nearly complete embolism, and <90% embolization as partial embolism. The incidence of complications such as hydrocephalus, rebleeding and cerebral vasospasm was observed. Cranial Doppler ultrasonography showed that cerebral vasospasm occurred when the blood flow velocity of the middle cerebral artery was >120 cm/s.

### Evaluation of prognosis

Six months after operation, the prognosis was evaluated using the Glasgow Outcome Scale (GOS). Death was scored 1-point, vegetative state with minimal responses was scored 2 points, clear consciousness with severe disability or no ability to live independently was scored 3 points, mild disability with abilities to live independently and work under protection was scored 4 points, and recovery to normal life with mild defect was considered good recovery and scored 5 points. 1-3 points indicated poor prognosis, and 4-5 points meant good prognosis.

### Statistical analysis

SPSS 16.0 software was used for statistical analysis. The count data were expressed as n (%) and examined by the chi-square (χ^2^) test. The measurement data were expressed as mean ± standard deviation (^-^x ± s). One-way ANOVA was employed for multigroup comparisons, and the independent t-test was performed between two groups if the difference was statistically significant. The paired *t*-test was conducted for intragroup comparisons at different time points. Logistic regression model was utilized to analyse the factors affecting prognosis. Receiver operating characteristic (ROC) curve was plotted to evaluate the prognostic value of serum S100B level. α=0.05 was taken as the test level, and P<0.05 indicated that a difference was statistically significant.

## Results

### Baseline clinical data

The age, gender ratio, smoking history, drinking history, hypertension history, location of ruptured aneurysm, aneurysm diameter and Hunt-Hess grade of the three groups had no significant differences (P<0.05) (Table 1).

**Table 1:** Baseline clinical data

Group	Age_(year, x ± s)	Gender [n (%)]		Location of ruptured aneurysm [n (%)]				Hunt-Hess grade [n (%)]		Aneurysm diameter (mm, x̅ ± s)	Smoking history [n (%)]	Drinking history [n (%)]	Hypertension history [n (%)]
		
		Male	Female	Vertebrobasilar artery	Middle cerebral artery	Anterior communicating artery	Posterior communicating artery	I + II	III + IV				
Early-stage (n=66)	57.46 ±5.93	31(46.97)	35(53.03)	11(16.67)	13(19.70)	20(30.30)	27(40.91)	18(27.27)	34(51.52)	13.15±2.47	20(30.30)	27(40.91)	18(27.27)
Middle-stage (n=95)	58.12 ±6.01	46(48.42)	49(51.58)	16(16.84)	19(20.00)	29(30.53)	38(40.00)	26(27.37)	51(53.68)	12.94±2.43	29(30.53)	38(40.00)	26(27.37)
Late-stage (n=68)	57.83 ±5.97	34(50.00)	34(50.00)	13(19.12)	15(22.06)	21(30.88)	25(36.76)	15(22.06)	37(54.41)	12.78±2.39	21(30.88)	25(36.76)	15(22.06)
F/χ^2^	0.684	0.123		0.501				0.124		0.781	0.005	0.274	0.695
P	0.392	0.940		0.998				0.940		0.363	0.997	0.872	0.707

### Serum indices

S100B, MCP-1 and CRP levels had no significant differences among the three groups before operation (P>0.05), but decreased 1 week after operation. The levels were the highest in the early-stage group and the lowest in the late-stage group, showing significant differences between any two groups (P<0.05) (Table 2).

**Table 2:** Serum indices

Group	S100B (µg/L, x̅ ± s)		MCP-1 (ng/L, x̅ ± s)		CRP (mg/L, x̅ ± s)	

	Before operation	1 week after operation	Before operation	1 week after operation	Before operation	1 week after operation
Early-stage (n=66)	3.05±0.36	0.82±0.10 ^a^	207.48±23.52	152.37±16.94 ^a^	47.25±5.18	26.39±2.85^a^
Middle-stage (n=95)	2.98±0.32	1.43±0.18 ^a^	206.91±22.87	173.45±18.62 ^a^	46.89±5.12	33.42±3.51^a^
Late-stage (n=68)	3.02±0.34	2.17±0.25 ^a^	207.16±23.05	189.61±20.75 ^a^	46.97±5.16	39.18±4.29^a^
F	1.087	26.495	0.153	5.247	0.468	10.142
P	0.143	0.000	0.898	0.000	0.573	0.000

a P<0.05 *vs.* before operation

### Clinical treatment outcomes

No significant differences were observed in the embolization effect or total incidence rate of complications between early- and middle-stage groups (P>0.05). The rate of complete embolization was higher and the total incidence rate of complications was lower in early- and middle-stage groups than those in the late-stage group (P<0.05). The GOS score was the highest in the early-stage group, and the lowest in the late-stage group, exhibiting significant differences between any two groups (P<0.05) (Table 3).

**Table 3:** Clinical treatment outcomes

Group	Embolization effect [n (%)]			Complications [n (%)]			GOS score (point, x̅ ± s)

	Complete	Nearly complete	Partial	Hydrocephalus	Rebleeding	Cerebral vasospasm	
Early-stage (n=66)	61(92.42)	4(6.06)	1(1.52)	2(3.03)	1(1.52)	1(1.52)	4.56 ±0.62
Middle-stage (n=95)	76(80.00)	12(12.63)	7(7.37)	8(8.42)	3(3.16)	2(2.11)	3.97 ±0.48
Late-stage (n=68)	40(58.82)	15(22.06)	13(19.12)	11(16.18)	4(5.88)	3(4.41)	3.63 ±0.41
F/χ^2^	23.368			11.096			5.768
P	0.000			0.004			0.000

### Univariate analysis results of factors affecting prognosis

The results of univariate analysis revealed that the patients with good and poor prognosis had significantly different age, aneurysm diameter, Hunt-Hess grade, timing of surgical treatment and levels of S100B, MCP-1 and CRP (P<0.05) (Table 4).

**Table 4:** Univariate analysis results of prognosis

Factor	Good prognosis (n=164)	Poor prognosis (n=65)	*t*/χ^2^	P
Gender [n (%)]			0.021	0.885
Male	79	32		
Female	85	33		
Age (year, x̅ ± s)	49.64±5.12	65.87 ±6.73	19.701	0.000
Aneurysm diameter (mm, x̅ ± s)	10.58±2.06	16.49±2.81	18.896	0.000
Location of ruptured aneurysm [n (%)]			5.316	0.150
Vertebrobasilar artery	28	12		
Middle cerebral artery	37	10		
Anterior communicating artery	61	19		
Posterior communicating artery	38	24		
Hunt-Hess grade upon admission [n (%)]			39.415	0.000
I + II	98	9		
III + IV	66	56		
Timing of operation [n (%)]			24.038	0.000
Early stage	59	7		
Middle stage	70	25		
Late stage	35	33		
Embolization effect [n (%)]			0.600	0.741
Complete	125	52		
Near complete	24	7		
Partial	15	6		
Postoperative hydrocephalus [n (%)]	15	6	0.000	0.984
Postoperative rebleeding [n (%)]	6	2	0.047	0.829
Postoperative cerebral vasospasm [n (%)]	5	1	0.416	0.519
Smoking history [n (%)]	49	21	0.130	0.719
Drinking history [n (%)]	67	23	0.584	0.445
Hypertension history [n (%)]	42	17	0.007	0.932
S100B (µg/L, x̅ ± s)	2.13±0.26	3.65±0.39	34.298	0.000
MCP-1(ng/L, x̅ ± s)	185.72±19.83	234.59±25.48	15.456	0.000
CRP (mg/L, x̅ ± s)	42.58±4.37	50.73±5.29	11.964	0.000

### Multivariate analysis results of factors affecting prognosis

Multivariate analysis exhibited that older age, large diameter of aneurysm, high Hunt-Hess grade upon admission, late-stage surgical treatment and high S100B level were risk factors for the poor prognosis of patients (Table 5).

**Table 5:** Multivariate analysis results of prognosis

Factor	β	SE	Wald	*P*	OR (95%CI)
Age	0.467	0.599	1.204	0.005	2.178(1.306~2.952)
Aneurysm diameter	0.581	0.673	1.316	0.017	2.952(2.183~3.645)
Hunt-Hess grade upon admission [n (%)]	0.736	0.912	2.027	0.009	3.285(1.592~4.036)
Timing of surgical treatment	0.675	0.834	2.185	0.013	1.639(1.245~2.163)
S100B	0.943	0.726	1.454	0.022	4.172(2.067~5.428)
MCP-1	0.328	0.457	1.539	0.346	1.089(0.528~1.639)
CRP	0.891	0.758	1.463	0.228	0.954(0.326~2.017)

Assignment of independent variables: Age: ≥60 years old=1, <60 years old=0; aneurysm diameter: ≥12 mm=l, <12 mm=0; Hunt-Hess grade upon admission: III + IV=1, I + II=0; timing of surgical treatment: late stage=1, early stage and middle stage=0; S100B: ≥3.1 µg/ L=1, <3.1 µg /L=0; MCP-1: ≥210 ng/L=1; <210 ng/L=0; CRP: ≥46 mg/L=1; <46 mg/L=0.

### Predictive value of S100B for prognosis

**Figure 1:**
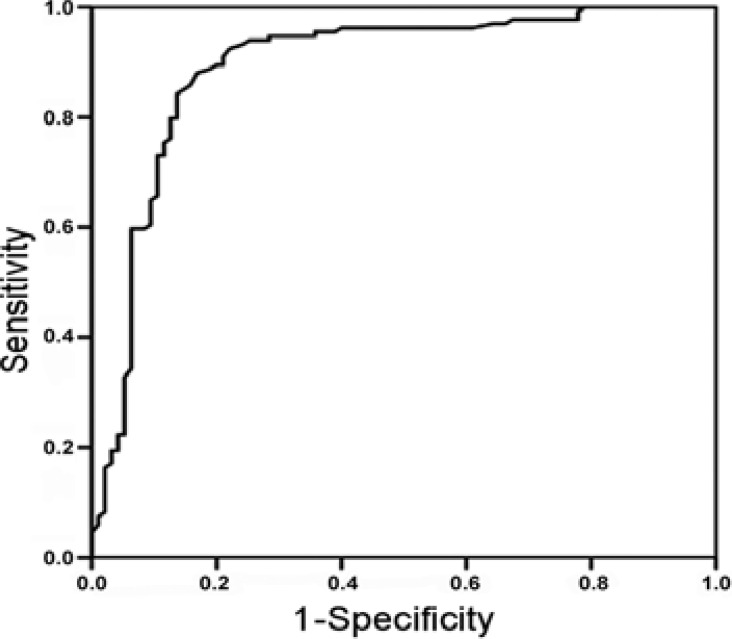
Predictive value of S100B for prognosis

The optimal cut-off value of S100B for predicting the prognosis of patients was 2.785 µg/L, and the area under the ROC curve, sensitivity, specificity, Youden index and 95% confidence interval were 0.892, 84.3%, 86.3%, 0.706 and 0.844-0.940, respectively (Figure 1).

## Discussion

In recent years, endovascular embolization has been extensively employed to treat aSAH, and verified to effectively prevent the growth and rupture of intracranial aneurysms^4^. Early interventional treatment has been recommended, because the best opportunity is frequently missed due to conservative treatment, which leads to a higher risk of cerebral vasospasm or recurrent aneurysm rupture and bleeding. Moreover, angiography causes great damage to the cerebral vessels of patients with severe conditions^5^. In contrast, late interventional treatment has also been supported, because the patients' physical function can be recovered and their tolerance is enhanced after sufficient conservative treatment, which is conducive to postoperative rehabilitation^6^. In this study, earlier vascular interventional embolization led to higher rate of complete embolization, lower total incidence rate of complications and better prognosis, being consistent with the beneficial role of interventional treatment in the early stage^5^. Probably, on one hand, with the development of interventional therapy, it is easier to place micro-catheter into aneurysm at the early stage, exerting good embolization effects. On the other hand, timely treatment promotes the recovery of neurological function.

Mainly distributed in Schwann cells and astrocytes of the central nervous system, S100B is a low-molecular-weight acidic calcium-binding protein that is released into the blood through the damaged blood-brain barrier, thus markedly elevating the concentration in the serum. Hence, serum S100B level is often utilized to reflect the integrity of the blood-brain barrier and the severity of brain tissue damage^7^. As an important inflammatory cytokine in human body, MCP-1 is secreted by neurons and macrophages during brain damage, which plays a chemotactic role on mononuclear macrophags and infiltrates into the brain parenchyma, thereby up-regulating the expressions of adhesion molecules to participate in the process of ischemic brain injury. Bontekoe *et al.^8^* found that the MCP-1 level plummeted after early- and late-stage interventional embolization, and it was evidently lower in the early-stage group than that in the late-stage group. Additionally, CRP is a common clinical index for evaluating inflammation and trauma. In this study, the serum levels of S100B, MCP-1 and CRP levels reduced 1 week after operation, which were the lowest in the early-stage group and the highest in the late-stage group. Besides, milder nerve defect was caused by brain tissue injury owing to earlier the interventional embolization, which was beneficial to the recovery of patients. The above findings were consistent with those in a previous literature^9^.

Rigante *et al.*^10^ reported that multiple aneurysms and older age were risk factors for the poor prognosis of aSAH patients. In addition, Weiland *et al.^11^* found that age, an-eurysm size and postoperative cerebral vasospasm significantly affected the prognosis of aSAH patients. In this study, older age, large diameter of aneurysm, high Hunt-Hess grade upon admission, late-stage surgical treatment and high S100B level were the risk factors for poor prognosis. The different findings may be attributed to the differences between inclusion criteria, sample size and regions of subjects. Similar to this study, Daou et al. reported that the probability of recurrent rupture and bleeding of aneurysms in patients aged above 70 years old was remarkably increased, and the neurological function of elderly patients declined faster after onset, with a higher mortality rate^12^. When intracranial aneurysm rupture

In conclusion, earlier vascular interventional embolization is conducive to the mitigation of brain tissue injury and the decrease of serum S100B level, leading to better treatment outcomes and prognosis. Therefore, various factors of patients should be evaluated timely and comprehensively, and vascular interventional embolization should be conducted as early as possible. Regardless, this study is still limited. This study has a small sample size, so the results may be biased. Further multicenter studies with larger sample sizes are ongoing in our group to validate the findings.
